# Combined Effect of Arginine, Valine, and Serine on Exercise-Induced Fatigue in Healthy Volunteers: A Randomized, Double-Blinded, Placebo-Controlled Crossover Study

**DOI:** 10.3390/nu11040862

**Published:** 2019-04-17

**Authors:** Yuichi Tsuda, Makoto Yamaguchi, Teruyuki Noma, Eiji Okaya, Hiroyuki Itoh

**Affiliations:** R&D Division, Meiji Co., Ltd., 1-29-1 Nanakuni, Hachiouji, Tokyo 192-0919, Japan; makoto.yamaguchi@meiji.com (M.Y.); teruyuki.noma@meiji.com (T.N.); eiji.okaya@meiji.com (E.O.); hiroyuki.itou@meiji.com (H.I.)

**Keywords:** amino acid mixture, fatigue, exercise, visual analog scale

## Abstract

Although several kinds of amino acids (AAs) are known to affect physiological actions during exercise, little is known about the combined effects of a mixture of several AAs on fatigue during exercise. The aim of the present study was to investigate the effect of an AA mixture supplement containing arginine, valine, and serine on exercise-induced fatigue in healthy volunteers. These AAs were selected because they were expected to reduce fatigue during exercise by acting the positive effects synergistically. A randomized, double-blinded, placebo-controlled crossover trial was conducted. Thirty-nine males ingested an AA mixture containing 3600 mg of arginine, 2200 mg of valine, and 200 mg of serine or a placebo each day for 14 days. On the 14th day, the participants completed an exercise trial on a cycle ergometer at 50% of VO_2_max for 120 min. After the two-week washout period, the participants repeated the same trial with the other test sample. The participant’s feeling of fatigue based on a visual analog scale (VAS) and a rating of perceived exertion (RPE), as well as blood and physical parameters were evaluated. The feeling of fatigue based on VAS and RPE were significantly improved in AA compared to those in placebo. In the blood analysis, the increase in serum total ketone bodies during exercise and plasma tryptophan/branched-chain amino acids were significantly lower in AA than those in placebo. The present study demonstrated that supplementation with an AA mixture containing arginine, valine, and serine reduced the feeling of fatigue during exercise. The AA mixture also changed several blood parameters, which may contribute to the anti-fatigue effect.

## 1. Introduction

Fatigue during exercise, which might induce decreased exercise performance or motivation, has been reported to be related to various biological factors, including metabolic factors and hormones. It was demonstrated that prolonged exercise induced a decrease in blood glucose, which caused an increase in the sense of fatigue [1] and that carbohydrate supplementation postponed fatigue during exercise by maintaining blood glucose levels [2,3]. One of the glucocorticoid hormones, cortisol, which quickly increases during exercise [4,5], was reported to significantly correlate with the feeling of fatigue during exercise [6,7]. It was also reported that ammonia, which is produced by accelerating the catabolism of muscle proteins when energy is depleted during prolonged exercise, led to the feeling of fatigue through neurotoxic effects [8,9].

Several kinds of amino acids (AAs) are known to affect physiological actions like the changes of blood or physical parameters during exercise. Arginine, known as a substrate of nitric oxide, significantly decreased exercise-induced blood ammonia and lactate elevation [10]. Moreover, several researchers have claimed that arginine supplementation increased exercise performance by regulating vasodilatation and blood flow [11,12,13]. In these studies, 1.5–9 g of arginine were supplemented for several days. Branched-chain amino acids (BCAAs) valine, leucine, and isoleucine are also known to have various effects on physiological actions during exercise, and a large number of studies have evaluated the effects of BCAA supplementation. For example, a BCAA mixture supplement (2.5 g) decreased the serum creatine kinase concentration following prolonged exercise, which suggested that BCAAs could reduce muscle damage associated with endurance exercise [14]. De Palo et al., reported that chronic supplementation with a BCAA mixture (9.64 g) suppressed the increase in plasma lactate concentration after exercise [15]. It was also reported that the intake of BCAAs increases their concentration in plasma and prevents the increase in the free tryptophan/BCAA ratio, which could decrease the synthesis of serotonin in the brain and delay central fatigue [16,17]. Some studies of a single kind of BCAA have shown that leucine accelerates protein synthesis [18,19] and that valine supplementation before exercise suppresses the elevation of corticosterone levels and the depletion of glucose during exercise in rats [20]. Serine, which is known as a substrate of phosphatidylserine, was reported to reduce the feeling of fatigue, improve exercise performance [21,22] and reduce the increase in cortisol during exercise [23]. In these reports, 400–750 mg of phosphatidylserine caused significant effects.

However, little is known about the combined effects of supplementation with mixtures of several AAs (except for the BCAA mixture), especially on the feeling of fatigue during exercise. In the present study, we developed an AA mixture containing arginine, valine, and serine, which had the above-described physiological effects related to the feeling of fatigue. At first, valine was selected because BCAAs may be able to delay central fatigue by decreasing the synthesis of serotonin in the brain [16,17]. Actually, Gomez-Merino et al., reported that pre-exercise administration of valine significantly prevented the exercise-induced serotonin release in the brain of a rat [24]. Leucine and isoleucine were not included because they were able to promote glucose uptake and utilization, which may deplete blood glucose or muscle glycogen and induce fatigue by leucine or isoleucine supplementation before exercise [20,25,26]. However, it was also reported that BCAA supplementation elevated ammonia accumulation during exercise [27]. Secondly, arginine was selected because arginine was expected to reduce the increase in plasma ammonia via increased ureagenesis [10] and increase exercise performance by regulating vasodilatation and blood flow [11,12,13]. In addition, serine was selected because serine may be able to decrease fatigue via different mechanism from valine or arginine at low doses. The dose of the developed AA mixture was set to be the minimum required amount which could be expected to produce the positive effects and was approximately the same as those in the other studies. The aim of the present study was to investigate the effect of supplementation with an AA mixture containing arginine, valine, and serine on exercise-induced fatigue in healthy volunteers by comparing with a placebo supplementation. We hypothesized that the AA mixture supplementation could reduce fatigue during exercise by producing the positive effects of arginine, valine, and serine synergistically.

## 2. Materials and Methods

### 2.1. Subjects

**Figure 1:**
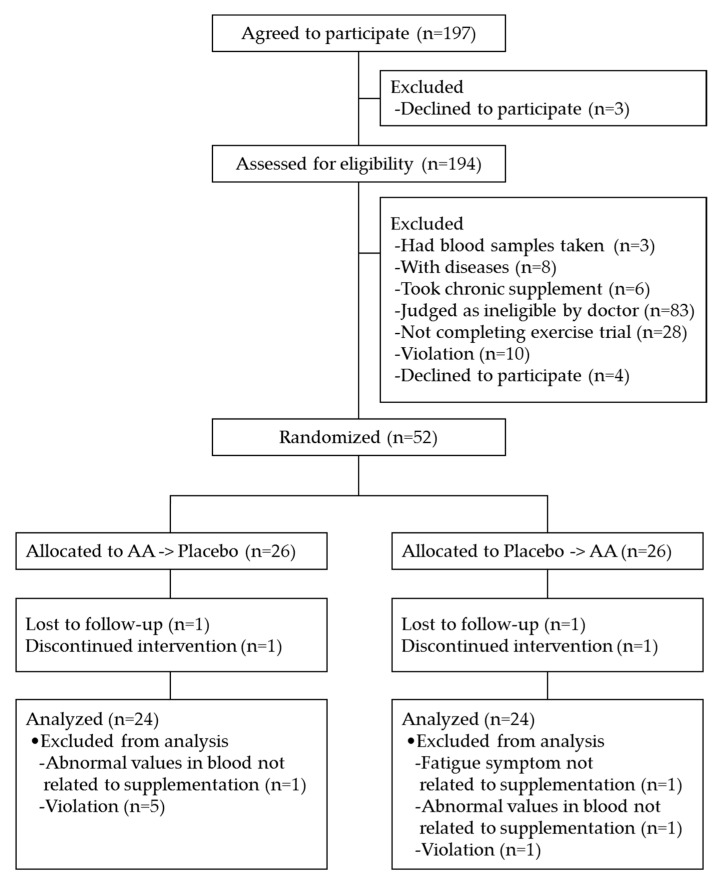
Flowchart of the study participants. AA: amino acid.

A total of 197 healthy male volunteers aged 30 to 59 years old were recruited from a volunteer database of Soiken Inc. (Osaka, Japan) and gave their written informed consent. Subjects who met the exclusion criteria (subjects with food allergies, subjects with a smoking habit, subjects who had blood samples of more than 200 mL or 400 mL taken within 1 month or 3 months prior to the start of the study, subjects who participated in other clinical studies within the prior month, subjects with any diseases that may affect the evaluation in the study, subjects who took chronic supplemental AAs, or subjects who were judged as ineligible by a doctor for other reasons) were excluded (a total of 110 subjects were excluded). Subjects were included if they could complete the exercise trial of this study (a total of 28 subjects who could not complete the exercise trial were excluded). The recruitment and selection of participants were conducted between March and May 2018. Seven subjects dropped out due to personal reasons before allocation, and a total of 52 subjects were randomly allocated into two groups in a 1:1 ratio using a computer-generated random number sequence by an investigator who had no contact with the subjects or researchers. The sequence allocation concealment and blinding of the subjects and researchers were maintained throughout the study. The sample size of subjects was selected to achieve 80% power at a 5% significance level taking into account that several subjects might have dropped out. In the study, four subjects dropped out due to personal reasons after allocation, and 48 subjects completed the entire protocol. Before the statistical analysis, one participant dropped out due to a subjective symptom of fatigue not related to the supplementation on the exercise trial day, two participants dropped out due to abnormal values in their blood analyses not related to the supplementation, and six dropped out due to violations of study compliance (vigorous exercise on the day before the exercise day or non-compliance of intake time of a provided meal). We, therefore, analyzed a final total of 39 subjects, see Figure 1. The basic characteristics of the study subjects are shown in Table 1. The subjects were instructed not to change their usual exercise volume or diet during the study. The subjects recorded their sleep time, alcohol intake, test supplement intake, amount of exercise, and medication every day during the study period. They were also instructed not to take alcohol or vigorous exercise from the day before the exercise trial day until the exercise trial.

### 2.2. Study Design

**Figure 2:**
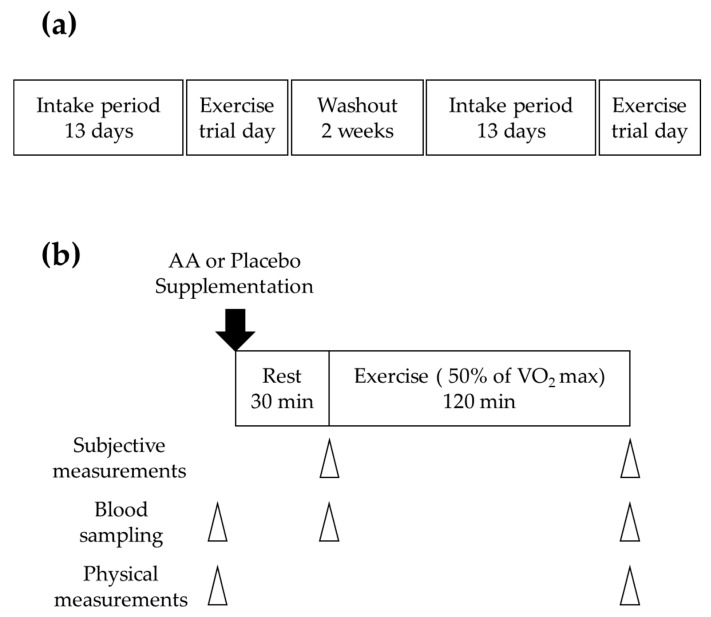
(**a**) Study design; (**b**) experimental procedure on the exercise trial day. AA: amino acid.

The study was a randomized, double-blinded, placebo-controlled crossover trial that was conducted at the Esaka Research Center (Soiken Inc., Osaka, Japan), see Figure 2a. All subjects participated in two trials with a 2-week washout period. The participants ingested one of the test samples (AA granule or placebo granule) for 14 days and carried out an exercise trial on the final day of supplementation. After the 2-week washout period, the participants repeated the same trial with the other test sample. These trials were conducted between June and July 2018. The study protocol was approved by the Institutional Review Board of Fukuda Clinic (Osaka, Japan) (Approval No. IRB-20180120-1) and the Meiji Institutional Review Board (Tokyo, Japan) (Approval No. 130) and was registered in the UMIN Clinical Trials Registry (UMIN000031150) on February 24, 2018. The study was conducted in accordance with the Declaration of Helsinki.

### 2.3. Experimental Procedure

Subjects were provided packets of AA granule containing 1800 mg of arginine, 1100 mg of valine, and 100 mg of serine (Kyowa Hakko Bio Co., Ltd., Tokyo, Japan) per packet (not containing carbohydrate) or packets of placebo granule, in which the AAs were replaced by almost the same amount of sucrose (not containing AAs), in a randomized order and in a double-blinded fashion. The two test samples were indistinguishable by appearance, and their tastes were also unidentifiable because they contain an acidulant, which masks the sweetness. Subjects ingested two packets per day for 13 days, and it was recommended that they should ingest the packets before exercise if they take exercise. On the 13th day, the participants had the same dinner. On the next day (the 14th day), they had the same breakfast (carbohydrate jelly) with one packet of the test sample, and they ingested an additional packet 30 min before the exercise trial. Then, they carried out an exercise trial on a cycle ergometer (Aerobike 75XLII or 75XLIII, Konami Sports Life Co., Ltd., Tokyo, Japan) at 50% of the individual’s maximal oxygen consumption (VO_2_max) for 120 min, see Figure 2b. The intensity and time of exercise were determined to sufficiently increase the subject’s feeling of fatigue due to exercise [27,28]. The exercise trial was conducted in a temperature- and humidity-controlled environment.

### 2.4. Subjective Measurements

Subjects were asked to subjectively rate their feeling of fatigue on a visual analog scale (VAS) from 0 (no fatigue) to 100 (total exhaustion) and on a Borg’s 6- to 20-point rating scale of perceived exertion (RPE) before and after the exercise. In addition, participants completed a Profile of Mood States-Short Form (POMS-SF) before and after the exercise, and we calculated the total mood disturbance score to assess their moods.

### 2.5. Blood Sampling

Blood samples (20 mL) were collected from the brachial vein after the visit to the research center, which means before the ingestion of a packet 30 min before the exercise trial, before the exercise, and after the exercise. Whole blood from a NaF- and EDTA-2Na-containing tube and an EDTA-2Na-containing tube were centrifuged immediately at 1700× *g* for 10 min at 4 °C, and the plasma was separated for analysis of glucose by a UV method using biochemical auto-analyzer Bio Majesty JCA-BM 9130 (JEOL Ltd., Tokyo, Japan) and analyses of AAs (aspartic acid, threonine, serine, asparagine, glutamic acid, glutamine, proline, glycine, alanine, citrulline, valine, cystine, methionine, isoleucine, leucine, tyrosine, phenylalanine, β-alanine, ornithine, tryptophan, lysine, histidine, and arginine) and ammonia by high-performance liquid chromatography using amino acid auto-analyzer model L-8900 (Hitachi High-Tech Science Corporation, Tokyo, Japan). Serum samples were prepared by collecting whole blood in a plain tube and centrifuging the blood at 1700× *g* for 10 min at 4 °C for analysis of the total ketone bodies by an enzyme cycling method using the biochemical auto-analyzer, Bio Majesty JCA-BM 8060 (JEOL Ltd., Tokyo, Japan). Cortisol was assessed by a chemiluminescence immunoassay using an ADVIA Centaur XP (Siemens Healthineers, Erlangen, Germany), and free fatty acids were analyzed by an enzymatic method using a biochemical auto-analyzer, Bio Majesty JCA-BM 8060. Whole blood was deproteinized in a 1 N perchloric acid-containing tube for 15–60 min on ice and was then centrifuged at 1700× *g* for 10 min at 4 °C for the lactate analysis by an enzymatic method using a biochemical auto-analyzer, Bio Majesty JCA-BM 9130 (JEOL Ltd., Tokyo, Japan). Assays of plasma glucose, AAs, ammonia, serum total ketone bodies, cortisol, and blood lactate were performed at BML, Inc. (Tokyo, Japan), and the analysis of serum free fatty acids was performed at Skylight Biotech Inc. (Tokyo, Japan).

### 2.6. Physical Measurements

Blood pressure, pulse rate, body temperature, body weight, and body fat percentage were measured after the visit to the research center and the exercise protocol. Blood pressure was evaluated by auscultation using an MMI-101 mercury sphygmomanometer (Muranaka Medical Instruments Co., Ltd., Osaka, Japan). Pulse rate and body temperature were assessed by a pulse oximetry ear sensor (Konami Sports Life Co., Ltd., Kanagawa, Japan) and an ear thermometer MC-510 (Omron Healthcare Co., Ltd., Kyoto, Japan). Body weight and body fat percentage were measured by an MC-780A body composition meter (Tanita Co., Ltd., Tokyo, Japan).

### 2.7. Statistical Analysis

In the study, the subjective measurement of the VAS was a primary outcome and the others were secondary outcomes. All data are expressed as the mean ± standard deviation (SD). Data were analyzed using a two-way repeated measures analysis of variance (ANOVA) with treatment (placebo and AA) and time (subjective measurements: before exercise and after exercise; blood parameters: after visit, before exercise, and after exercise; physical parameters: after visit and after exercise). Significant main effects and interactions were explored with a Bonferroni-corrected post-hoc *t*-test. The change scores before and after exercise were analyzed using a Student’s *t*-test for the comparisons of pairs of groups. Analyses were performed with SPSS v. 22 (IBM Japan, Ltd., Tokyo, Japan). Differences with *p*-values < 0.05 were considered significant.

## 3. Results

No subjects reported any side effects related to the supplementation, and there was no significant treatment order effect in the study. There were no differences between the AA group and the placebo group on their sleep time, alcohol intake, test supplement intake, amount of exercise, and medication.

### 3.1. Subjective Measurements

A two-way repeated measures ANOVA (treatment × time) was conducted for the feeling of fatigue on the VAS and there were significant predominant effects for treatment (*p* = 0.006) and time (*p* < 0.001). There was no significant interaction between treatment and time (*p* = 0.132). A subsequent Bonferroni-corrected post-hoc t-test demonstrated that the VAS score after the exercise was significantly lower in the AA group than that of the placebo group (58.2 ± 16.5 vs. 62.6 ± 15.7, *p* = 0.010), see Table 2. In addition, there were significant predominant effects for treatment (*p* = 0.046) and time (*p* < 0.001), and there was no significant interaction between treatment and time (*p* = 0.132) on the RPE. A post-hoc *t*-test showed that the RPE improved significantly in the AA group compared to that in the placebo group before the exercise (7.7 ± 1.5 vs. 8.1 ± 1.5, *p* = 0.040), see Table 3. A two-way repeated measures ANOVA for the total mood disturbance scores of the POMS-SF revealed no significant predominant effect for treatment or interaction between treatment and time (before the exercise: AA 5.2 ± 12.4 vs. placebo 5.2 ± 13.8; after the exercise: AA 6.7 ± 12.6 vs. placebo 6.1 ± 12.8).

### 3.2. Blood Analysis

A two-way repeated measures ANOVA (treatment × time) for plasma glucose, blood lactate, serum free fatty acid, serum total ketone bodies, and serum cortisol revealed that no significant predominant effects for treatment were present. There was a significant interaction between treatment and time on serum total ketone bodies (*p* = 0.034). While a post-hoc *t*-test showed no significant difference between the AA group and the placebo group, the AA mixture supplement had a tendency to increase the serum total ketone body levels before exercise compared to the placebo (*p* = 0.053). In addition, the increase in serum total ketone bodies between before and after the exercise was significantly lower in the AA group than that in the placebo group (*p* < 0.05), see Table 4. Plasma AA concentrations are displayed in Table 5. There were significant interactions between treatment and time on the levels of arginine (*p* < 0.001), valine (*p* < 0.001), serine (*p* = 0.003), citrulline (*p* = 0.004), ornithine (*p* < 0.001), and tryptophan/BCAA ratio (*p* < 0.001). A post-hoc *t*-test revealed that the intake of the AA mixture significantly increased the levels of arginine, valine, citrulline, and ornithine and decreased the level of tryptophan/BCAA ratio compared to the placebo after the visit, before the exercise, and after the exercise (*p* < 0.01). The plasma serine concentration before exercise was also significantly higher in the AA group than that in the placebo group (*p* < 0.01). There was no significant main effect for treatment or interaction between treatment and time on plasma ammonia level.

### 3.3. Physical Measurements

A two-way repeated measures ANOVA (treatment × time) was conducted for the physical parameters and there were no significant predominant effects for treatment or interactions between treatment and time, see Table 6.

## 4. Discussion

The present study aimed to investigate the effect of combined arginine, valine, and serine supplementation on fatigue during exercise in healthy volunteers. The VAS and RPE, which were used for evaluating the feeling of fatigue during exercise in the study, have been shown to be valid instruments for the quantitative assessment of fatigue [29,30]. In the study, there were significant predominant effects for treatment on VAS and RPE. Moreover, the feeling of fatigue on the VAS after the exercise was significantly lower in the AA group than that in the placebo group, which indicated that supplementation with the AA mixture containing arginine, valine, and serine markedly reduced exercise-induced fatigue. The RPE score was significantly lower in the AA group than that in the placebo group before the exercise, which suggested that the AA mixture used in the present study improved the condition of subjects before the exercise. The result may indicate that the AA mixture could reduce the sense of fatigue induced by physical activities in daily life.

Ketone bodies are lipid-derived organic compounds that can serve as a circulating energy source for tissues such as brain or skeletal muscle tissue in the fasting state or during prolonged exercise [31]. Johnson et al., reported that well-trained individuals demonstrated an attenuated rise in plasma ketone body concentrations during and after exercise compared to untrained individuals [32]. Some reports have claimed that ketone bodies enhance exercise performance via modulation of energy metabolism or decreases in central (i.e., neural brain) fatigue [33,34,35]. In the study, there was a significant interaction between treatment and time on serum total ketone bodies, and the AA mixture supplement had a tendency to increase the serum total ketone body levels before exercise compared to the placebo, which suggested that the AA supplement might contribute to enhance the production of the ketone bodies. It has been reported that arginine stimulates the secretion of glucagon from pancreatic alpha cells [36], which is known to promote lipolysis. Therefore, the tendency of the total ketone bodies in the study to increase might have been caused by arginine-stimulated glucagon secretion. On the other hand, the change in total ketone bodies during exercise was significantly lower in the AA group than that in the placebo group. These data indicated that the increased ketone bodies were effectively used during exercise and that the AA mixture supplement might accelerate the utilization of the ketone bodies during the exercise trial. The promotion of the utilization of ketone bodies may be related to the anti-fatigue effect during exercise that was associated with supplementation with the AA mixture in the study.

The AA mixture supplement significantly increased the levels of citrulline, which is produced via the urea cycle [37] after the visit, before the exercise, and after the exercise. Supplementation with citrulline, a potent precursor of arginine, was reported to enhance cycling time trial performance and improve subjective feelings (e.g., of muscle soreness) after performance through its effect on nitric oxide synthesis [38], which indicated that the increase in citrulline concentrations may be one of the factors related to the attenuation of exercise-induced fatigue by the AA mixture in the study.

Fernstrom et al., showed that exercise-induced increases in neuronal serotonin synthesis and release may contribute to the development of central fatigue [39]. In fact, it was revealed that exercise significantly increased the level of brain tryptophan, the precursor for cerebral serotonin synthesis, and the brain serotonin concentration in a rodent study [16]. It was also reported that the transport of tryptophan across the blood–brain barrier is influenced by the concentration of BCAAs, which are transported via the same carrier system [40,41,42]. This indicates that the intake of BCAAs increases their concentration in plasma and decreases the synthesis of serotonin in the brain by competing with tryptophan for the transporter to cross the blood—brain barrier, which means that preventing the increase in the free tryptophan/BCAA ratio might be able to delay central fatigue [16,17]. In this study, the plasma valine concentration was significantly higher in the AA group while there were no differences in the levels of plasma leucine and isoleucine between the AA group and the placebo group. These data demonstrated that the increase of valine did not affect leucine and isoleucine concentrations. Furthermore, the level of the tryptophan/BCAA ratio significantly decreased in the AA group. The result suggested that the AA mixture supplementation could attenuate tryptophan transport into the brain and the synthesis of serotonin in the brain, which may have caused a decrease in fatigue during exercise in the study. Although citrulline is known to enhance the use of BCAAs [38,43], which could induce the depletion of plasma BCAAs and central fatigue via neuronal serotonin synthesis, the AA mixture used in this study could negate the negative effect of the increase of citrulline by increasing the plasma valine concentration.

Ammonia is produced by the catabolism of muscle proteins when energy is depleted during exercise, which is reported to cause the feeling of fatigue through neurotoxic effects [8,9]. It was also reported that BCAA supplementation elevated ammonia accumulation during exercise [27]. On the other hand, Schaefer et al., revealed that arginine reduced the exercise-induced increase in plasma ammonia via increased ureagenesis [10] and it was reported that ornithine administration could attenuate physical fatigue by increasing the efficiency of energy consumption and promoting the excretion of ammonia [28]. In the study, there was no significant effect on the level of ammonia by the AA mixture supplementation even though the AA mixture contained valine, which might be caused by the significant increases of arginine and ornithine. The ammonia-clearance effects of arginine and ornithine may be superior to the ammonia-accumulation effect of valine in the AA mixture and could contribute to prevent from increasing fatigue.

The level of serine before exercise was also significantly increased and serine concentration was significantly decreased during exercise in the AA group. These data indicated that the use of serine was enhanced in the AA group in the study. It was reported that supplementation with phosphatidylserine, synthesized by serine, attenuated the perception of fatigue during exercise and improved exercise capacity [21,22], which suggested that the increase in serine might contribute to the phosphatidylserine production that affected the feeling of fatigue in the study.

There were some limitations in the present study. At first, we did not conduct performance tests such as a time trial test or Wingate test in the study. Further studies should be conducted to clarify whether the anti-fatigue effect of the AA mixture could improve exercise performance. In addition, we did not evaluate the food intake of the subjects during the study except for the dinner they consumed the day before the test day and their breakfast on the test day. Although the subjects claimed to have adhered to the instructions, the lack of information on daily energy intake or macronutrient composition limits the evaluation of whether these factors influenced the effect of the AA mixture supplement. Analyzing the dietary information of the subjects will give more accurate results in future studies. Moreover, the blood parameters evaluated in the study could not directly indicate the promotion of ketone body utilization during exercise or the suppression of the synthesis of serotonin in the brain. Further investigations measuring the effect of the AA mixture supplementation on the enzyme activities related to the ketone body metabolism or the serotonin synthesis in the brain using a rodent study will support the result of this study.

## 5. Conclusions

In conclusion, this study demonstrated that supplementation with an AA mixture containing arginine, valine, and serine reduced the feeling of fatigue during exercise, which may be caused by the combined effect of the promotion of ketone body utilization, the suppression of the synthesis of serotonin in the brain, the increase of some amino acids related to fatigue, and the elimination of ammonia.

## Figures and Tables

**Table 1:** Basic characteristics of the study subjects.

	AA -> Placebo	Placebo -> AA
	*n* = 18	*n* = 21
Age (years)	50.6 ± 6.5	49.8 ± 5.9
Height (cm)	168.8 ± 6.3	170.3 ± 6.0
Body weight (kg)	65.8 ± 6.9	68.8 ± 8.7
VO_2_ max (mL/min/kg)	34.7 ± 5.6	32.1 ± 4.5

Values are presented as the mean ± standard deviation (SD). There were no significant differences between two groups on basic characteristics. AA: amino acid.

**Table 2:** Visual analog scale (VAS) score before and after exercise.

	Before Exercise	After Exercise	*p*-Value for Interaction	*p*-Value for Treatment	*p*-Value for Time
AA	18.8 ± 11.4	58.2 ± 16.5 *	0.132	0.006	<0.001
Placebo	21.0 ± 12.5	62.6 ± 15.7			
Difference (95% CI)	−2.2 (−4.6, 0.1)	−4.4 (−7.4, −1.4)			

Values are mean ± SD (*n* = 39). AA: amino acid; CI: confidence interval. * *p* < 0.05 compared to the placebo group.

**Table 3:** Rating of perceived exertion (RPE) score before and after exercise.

	Before Exercise	After Exercise	*p*-Value for Interaction	*p*-Value for Treatment	*p*-Value for Time
AA	7.7 ± 1.5 *	13.7 ± 2.0	0.501	0.046	<0.001
Placebo	8.1 ± 1.5	14.0 ± 2.3			
Difference (95% CI)	−0.5 (−0.8, −0.1)	−0.2 (−0.8, 0.3)			

Values are mean ± SD (*n* = 39). AA: amino acid; CI: confidence interval. * *p* < 0.05 compared to the placebo group.

**Table 4:** Effects of the AA mixture supplement on blood biological parameters.

		After Visit	Before Exercise	After Exercise	Change before and after Exercise (95% CI)	*p*-Value for Interaction	*p*-Value for Treatment	*p*-Value for Time
Plasma glucose (mg/dL)	AA	88.8 ± 9.6	89.6 ± 6.6	80.4 ± 8.1	−9.2 (−11.9, −6.5)	0.454	0.323	<0.001
	Placebo	87.2 ± 9.1	89.7 ± 7.3	79.9 ± 6.4	−9.7 (−11.7, −7.7)			
Blood lactate (mg/dL)	AA	8.8 ± 3.1	7.8 ± 4.4	11.5 ± 3.6	3.7 (2.1, 5.3)	0.531	0.191	<0.001
	Placebo	9.7 ± 3.6	8.3 ± 2.9	11.7 ± 3.6	3.4 (2.1, 4.7)			
Serum free fatty acid (mEq/L)	AA	0.49 ± 0.21	0.42 ± 0.18	1.62 ± 0.43	1.20 (1.07, 1.33)	0.210	0.841	<0.001
	Placebo	0.49 ± 0.27	0.38 ± 0.18	1.68 ± 0.41	1.29 (1.15, 1.44)			
Serum total ketone body (µmol/L)	AA	172 ± 163	101 ± 85	582 ± 242	481 (419, 543) *	0.034	0.739	<0.001
	Placebo	134 ± 135	72 ± 53	630 ± 255	559 (479, 638)			
Serum cortisol (µg/dL)	AA	11.9 ± 3.9	10.9 ± 3.5	16.7 ± 6.3	5.8 (3.7, 7.9)	0.644	0.672	<0.001
	Placebo	12.2 ± 4.1	10.7 ± 3.6	17.3 ± 6.7	6.6 (4.0, 9.2)			

Values are mean ± SD (*n* = 39). AA: amino acid; CI: confidence interval. * *p* < 0.05 compared to the placebo group.

**Table 5:** Effects of the AA mixture supplement on amino acid concentrations.

		After Visit	Before Exercise	After Exercise	Change before and after Exercise (95% CI)	*p*-Value for Interaction	*p*-Value for Treatment	*p*-Value for Time
Aspartic acid (nmol/mL)	AA	3.8 ± 1.2 **	3.7 ± 1.3	3.5 ± 1.2	−0.1 (−0.6, 0.3)	0.027	0.379	<0.001
	Placebo	4.3 ± 1.2	3.5 ± 1.1	3.6 ± 1.2	0.1 (−0.2, 0.5)			
Threonine (nmol/mL)	AA	119.9 ± 22.5 **	125.5 ± 25.4	114.3 ± 22.1 **	−11.2 (−17.6, −4.8) *	0.010	<0.001	0.008
	Placebo	135.0 ± 26.2	128.8 ± 19.4	128.0 ± 21.8	−0.8 (−6.2, 4.6)			
Serine (nmol/mL)	AA	123.2 ± 25.2	128.3 ± 23.5 **	115.3 ± 17.3	−13.0 (−18.9, −7.0) **	0.003	0.657	0.001
	Placebo	126.4 ± 27.6	118.9 ± 20.5	118.6 ± 19.6	−0.3 (−5.7, 5.0)			
Asparagine (nmol/mL)	AA	48.0 ± 8.4	49.9 ± 8.8	45.9 ± 6.2	−4.0 (−6.6, −1.4)	0.234	0.376	0.030
	Placebo	49.5 ± 10.2	49.1 ± 7.4	47.9 ± 8.3	−1.2 (−3.7, 1.3)			
Glutamic acid (nmol/mL)	AA	62.2 ± 23.6	54.2 ± 21.3	55.9 ± 22.2	1.7 (−4.1, 7.5)	0.146	0.254	<0.001
	Placebo	67.7 ± 21.4	52.7 ± 17.7	58.4 ± 18.7	5.7 (0.9, 10.6)			
Glutamine (nmol/mL)	AA	632.8 ± 91.6	675.0 ± 107.5	648.8 ± 67.2	−26.2 (−57.2, 4.8)	0.105	0.911	0.038
	Placebo	643.2 ± 98.1	648.4 ± 56.3	667.9 ± 76.5	19.5 (−5.4, 44.4)			
Proline (nmol/mL)	AA	151.2 ± 58.8	148.3 ± 49.3	142.6 ± 41.7	−5.7 (−13.1, 1.7)	0.214	0.233	0.004
	Placebo	158.3 ± 46.0	147.9 ± 38.5	147.4 ± 40.1	−0.5 (−6.2, 5.3)			
Glycine (nmol/mL)	AA	250.4 ± 53.6	247.3 ± 52.4	227.2 ± 45.1 **	−20.2 (−31.0, −9.4)	0.383	0.020	<0.001
	Placebo	261.8 ± 64.8	251.9 ± 54.2	244.2 ± 54.1	−7.7 (−21.7, 6.2)			
Alanine (nmol/mL)	AA	420.9 ± 75.6	426.2 ± 85.5	401.2 ± 66.2	−25.0 (−49.9, −0.1)	0.174	0.174	0.009
	Placebo	425.7 ± 89.8	406.5 ± 69.5	386.3 ± 66.0	−20.2 (−41.5, 1.1)			
Citrulline (nmol/mL)	AA	42.6 ± 8.8 **	44.5 ± 9.8 **	51.6 ± 9.2 **	7.1 (4.2, 10.1)	0.004	<0.001	<0.001
	Placebo	38.2 ± 7.9	34.3 ± 6.3	43.9 ± 6.8	9.6 (7.8, 11.4)			
Valine (nmol/mL)	AA	488.3 ± 121.6 **	805.7 ± 190.1 **	596.1 ± 102.4 **	−209.6 (−263.4, −155.8) **	<0.001	<0.001	<0.001
	Placebo	242.0 ± 53.4	253.6 ± 114.5	243.7 ± 37.8	−9.9 (−48.9, 29.2)			
Cystine (nmol/mL)	AA	32.6 ± 7.8	31.9 ± 7.3	33.3 ± 6.3	1.4 (−0.4, 3.2)	0.903	0.823	0.079
	Placebo	32.7 ± 7.7	31.4 ± 6.1	33.1 ± 6.4	1.7 (0.3, 3.1)			
Methionine (nmol/mL)	AA	22.6 ± 3.8 **	22.9 ± 4.0	25.8 ± 3.4 **	2.9 (1.6, 4.3) *	0.048	<0.001	<0.001
	Placebo	24.6 ± 5.5	23.5 ± 3.2	28.4 ± 3.7	4.9 (3.8, 6.1)			
Isoleucine (nmol/mL)	AA	58.0 ± 11.6	59.9 ± 13.9	68.1 ± 12.4	8.2 (4.2, 12.2)	0.400	0.436	<0.001
	Placebo	60.1 ± 17.2	59.3 ± 11.9	69.6 ± 10.9	10.3 (7.2, 13.3)			
Leucine (nmol/mL)	AA	123.0 ± 20.3	127.2 ± 26.6	140.6 ± 24.7	13.4 (5.4, 21.4)	0.292	0.366	<0.001
	Placebo	123.8 ± 28.5	121.3 ± 20.8	138.7 ± 19.3	17.4 (11.6, 23.1)			
Tyrosine (nmol/mL)	AA	54.7 ± 11.7 **	54.8 ± 11.3	62.1 ± 10.4 **	7.3 (4.1, 10.6) *	0.079	<0.001	<0.001
	Placebo	60.6 ± 15.2	57.3 ± 11.3	68.9 ± 10.1	11.7 (9.0, 14.4)			
Phenylalanine (nmol/mL)	AA	59.4 ± 9.2	60.9 ± 11.2	64.9 ± 8.3 *	4.0 (0.0, 7.9)	0.285	0.040	<0.001
	Placebo	61.3 ± 9.5	61.1 ± 7.2	68.7 ± 7.5	7.5 (4.9, 10.1)			
β-Alanine (nmol/mL)	AA	2.6 ± 1.3	3.1 ± 1.8 **	3.2 ± 1.5 **	0.1 (−0.6, 0.7)	0.545	<0.001	0.043
	Placebo	2.2 ± 1.0	2.3 ± 1.2	2.5 ± 1.0	0.2 (−0.2, 0.5)			
Ornithine (nmol/mL)	AA	86.7 ± 22.9 **	107.1 ± 35.2 **	100.7 ± 21.2 **	−6.3 (−16.3, 3.6)	<0.001	<0.001	0.003
	Placebo	60.1 ± 16.0	58.0 ± 17.9	54.4 ± 10.1	−3.6 (−9.1, 1.9)			
Tryptophan (nmol/mL)	AA	38.1 ± 6.9	39.3 ± 8.6	28.0 ± 5.0	−11.4 (−13.9, −8.8)	0.481	0.257	<0.001
	Placebo	39.8 ± 8.9	39.3 ± 5.3	29.0 ± 6.3	−10.3 (−12.2, −8.4)			
Lysine (nmol/mL)	AA	188.9 ± 29.2 **	197.8 ± 40.3	188.6 ± 28.5 **	−9.2 (−19.8, 1.4) **	0.005	<0.001	0.914
	Placebo	213.4 ± 40.5	201.6 ± 30.2	212.8 ± 28.5	11.2 (2.8, 19.6)			
Histidine (nmol/mL)	AA	91.0 ± 13.3	92.9 ± 16.5	89.0 ± 12.4	−3.9 (−8.3, 0.5) *	0.095	0.573	0.317
	Placebo	94.1 ± 17.0	89.6 ± 9.7	91.7 ± 13.2	2.1 (−1.5, 5.7)			
Arginine (nmol/mL)	AA	144.5 ± 35.6 **	217.1 ± 55.2 **	168.9 ± 30.3 **	−48.2 (−64.0, −32.4) **	<0.001	<0.001	<0.001
	Placebo	94.5 ± 22.8	95.6 ± 26.7	100.4 ± 17.8	4.8 (−2.7, 12.3)			
Ammonia (nmol/mL)	AA	58.8 ± 12.9	56.4 ± 18.1	60.5 ± 14.3	4.0 (−0.8, 8.9)	0.292	0.181	0.004
	Placebo	63.3 ± 13.6	56.1 ± 12.0	63.3 ± 13.5	7.2 (2.6, 11.8)			
Tryptophan/BCAA	AA	0.060 ± 0.019 **	0.041 ± 0.013 **	0.035 ± 0.008 **	−0.006 (−0.010, −0.002) **	<0.001	<0.001	<0.001
	Placebo	0.095 ± 0.019	0.095 ± 0.020	0.065 ± 0.013	−0.030 (−0.035, −0.025)			

Values are mean ± SD (*n* = 39). AA: amino acid; CI: confidence interval. ** *p* < 0.01, * *p* < 0.05 compared to the placebo group.

**Table 6:** Effects of the AA mixture supplement on physical parameters.

		After Visit	After Exercise	*p*-Value for Interaction	*p*-Value for Treatment	*p*-Value for Time
Systolic blood pressure (mmHg)	AA	111.7 ± 9.4	112.3 ± 9.9	0.075	0.144	0.673
	Placebo	114.2 ± 10.8	112.6 ± 10.3			
Diastolic blood pressure (mmHg)	AA	73.9 ± 7.3	71.5 ± 9.1	0.850	0.427	0.022
	Placebo	74.4 ± 7.7	72.3 ± 9.0			
Pulse rate (bpm)	AA	70.5 ± 9.4	95.5 ± 13.6	0.298	0.619	<0.001
	Placebo	71.0 ± 8.3	93.8 ± 13.6			
Body temperature (°C)	AA	36.1 ± 0.4	36.0 ± 0.4	0.147	0.551	0.780
	Placebo	36.1 ± 0.4	36.1 ± 0.6			
Body weight (kg)	AA	65.8 ± 7.7	65.7 ± 7.6	0.670	0.216	<0.001
	Placebo	66.0 ± 7.7	65.8 ± 7.6			
Body fat (%)	AA	17.7 ± 4.5	18.4 ± 4.3	0.398	0.345	<0.001
	Placebo	17.6 ± 4.7	18.3 ± 4.6			

Values are presented as the mean ± SD (*n* = 39). AA: amino acid.

## References

[1] Grego F., Vallier J.-M., Collardeau M., Bermon S., Ferrari P., Candito M., Bayer P., Magnié M.-N., Brisswalter J. (2004). Effects of long duration exercise on cognitive function, blood glucose, and counterregulatory hormones in male cyclists. Neurosci. Lett..

[2] Coyle E., Hagberg J., Hurley B., Martin W., Ehsani A., Holloszy J. (1983). Carbohydrate feeding during prolonged strenuous exercise can delay fatigue. J. Appl. Physiol..

[3] Coyle E.F., Coggan A.R., Hemmert M., Ivy J.L. (1986). Muscle glycogen utilization during prolonged strenuous exercise when fed carbohydrate. J. Appl. Physiol..

[4] Jacks D.E., Sowash J., Anning J., Mcgloughlin T., Andres F. (2002). Effect of exercise at three exercise intensities on salivary cortisol. J. Strength Cond. Res..

[5] Hill E., Zack E., Battaglini C., Viru M., Viru A., Hackney A. (2008). Exercise and circulating cortisol levels: The intensity threshold effect. J. Endocrinol. Investig..

[6] McGuigan M.R., Egan A.D., Foster C. (2004). Salivary cortisol responses and perceived exertion during high intensity and low intensity bouts of resistance exercise. J. Sports Sci. Med..

[7] Caetano Júnior P., Castilho M., Raniero L. (2017). Salivary cortisol responses and session ratings of perceived exertion to a rugby match and fatigue test. Percept. Mot. Skills.

[8] Mutch B., Banister E. (1983). Ammonia metabolism in exercise and fatigue: A review. Med. Sci. Sports Exerc..

[9] Banister E., Cameron B. (1990). Exercise-induced hyperammonemia: Peripheral and central effects. Int. J. Sports Med..

[10] Schaefer A., Piquard F., Geny B., Doutreleau S., Lampert E., Mettauer B., Lonsdorfer J. (2002). L-arginine reduces exercise-induced increase in plasma lactate and ammonia. Int. J. Sports Med..

[11] Camic C.L., Housh T.J., Zuniga J.M., Hendrix R.C., Mielke M., Johnson G.O., Schmidt R.J. (2010). Effects of arginine-based supplements on the physical working capacity at the fatigue threshold. J. Strength Cond. Res..

[12] Bednarz B., Jaxa-Chamiec T., Gębalska J., Herbaczyńska-Cedro K., Ceremużyński L. (2004). L-arginine supplementation prolongs duration of exercise in congestive heart failure. Kardiol. Pol. (Pol. Heart J.).

[13] Bailey S.J., Winyard P.G., Vanhatalo A., Blackwell J.R., DiMenna F.J., Wilkerson D.P., Jones A.M. (2010). Acute l-arginine supplementation reduces the O_2_ cost of moderate-intensity exercise and enhances high-intensity exercise tolerance. J. Appl. Physiol..

[14] Greer B.K., Woodard J.L., White J.P., Arguello E.M., Haymes E.M. (2007). Branched-chain amino acid supplementation and indicators of muscle damage after endurance exercise. Int. J. Sport Nutr. Exerc. Metab..

[15] De Palo E., Gatti R., Cappellin E., Schiraldi C., De Palo C., Spinella P. (2001). Plasma lactate, gh and gh-binding protein levels in exercise following bcaa supplementation in athletes. Amino. Acids.

[16] Blomstrand E. (2006). A role for branched-chain amino acids in reducing central fatigue. J. Nutr..

[17] Newsholme E.A., Blomstrand E. (2006). Branched-chain amino acids and central fatigue. J. Nutr..

[18] Crozier S.J., Kimball S.R., Emmert S.W., Anthony J.C., Jefferson L.S. (2005). Oral leucine administration stimulates protein synthesis in rat skeletal muscle. J. Nutr..

[19] Anthony J.C., Yoshizawa F., Anthony T.G., Vary T.C., Jefferson L.S., Kimball S.R. (2000). Leucine stimulates translation initiation in skeletal muscle of postabsorptive rats via a rapamycin-sensitive pathway. J. Nutr..

[20] Tsuda Y., Iwasawa K., Yamaguchi M. (2018). Acute supplementation of valine reduces fatigue during swimming exercise in rats. Biosci. Biotechnol. Biochem..

[21] Wells A.J., Hoffman J.R., Gonzalez A.M., Stout J.R., Fragala M.S., Mangine G.T., McCormack W.P., Jajtner A.R., Townsend J.R., Robinson IV E.H. (2013). Phosphatidylserine and caffeine attenuate postexercise mood disturbance and perception of fatigue in humans. Nutr. Res..

[22] Kingsley M.I., Miller M., Kilduff L.P., McENENY J., Benton D. (2006). Effects of phosphatidylserine on exercise capacity during cycling in active males. Med. Sci. Sports Exerc.

[23] Starks M.A., Starks S.L., Kingsley M., Purpura M., Jäger R. (2008). The effects of phosphatidylserine on endocrine response to moderate intensity exercise. J. Int. Soc. Sports Nutr..

[24] Gomez-Merino D., Bequet F., Berthelot M., Riverain S., Chennaoui M., Guezennec C. (2001). Evidence that the branched-chain amino acid l-valine prevents exercise-induced release of 5-ht in rat hippocampus. Int. J. Sports Med..

[25] Nishitani S., Matsumura T., Fujitani S., Sonaka I., Miura Y., Yagasaki K. (2002). Leucine promotes glucose uptake in skeletal muscles of rats. Biochem. Biophys. Res. Commun..

[26] Doi M., Yamaoka I., Fukunaga T., Nakayama M. (2003). Isoleucine, a potent plasma glucose-lowering amino acid, stimulates glucose uptake in c2c12 myotubes. Biochem. Biophys. Res. Commun..

[27] Watson P., Shirreffs S.M., Maughan R.J. (2004). The effect of acute branched-chain amino acid supplementation on prolonged exercise capacity in a warm environment. Eur. J. Appl. Physiol..

[28] Sugino T., Shirai T., Kajimoto Y., Kajimoto O. (2008). L-ornithine supplementation attenuates physical fatigue in healthy volunteers by modulating lipid and amino acid metabolism. Nutr. Res..

[29] Lee K.A., Hicks G., Nino-Murcia G. (1991). Validity and reliability of a scale to assess fatigue. Psychiatry Res..

[30] Crewe H., Tucker R., Noakes T.D. (2008). The rate of increase in rating of perceived exertion predicts the duration of exercise to fatigue at a fixed power output in different environmental conditions. Eur. J. Appl. Physiol..

[31] Newman J.C., Verdin E. (2014). Ketone bodies as signaling metabolites. Trends Endocrinol. Metab..

[32] Winder W.W., Holloszy J.O., Baldwin K.M. (1974). Enzymes involved in ketone utilization in different types of muscle: Adaptation to exercise. Eur. J. Biochem..

[33] Cox P.J., Clarke K. (2014). Acute nutritional ketosis: Implications for exercise performance and metabolism. Extrem. Physiol. Med..

[34] Volek J.S., Noakes T., Phinney S.D. (2015). Rethinking fat as a fuel for endurance exercise. Eur. J. Sport Sci..

[35] Pinckaers P.J., Churchward-Venne T.A., Bailey D., van Loon L.J. (2017). Ketone bodies and exercise performance: The next magic bullet or merely hype?. Sports Med..

[36] Gerich J.E., Charles M.A., Grodsky G.M. (1974). Characterization of the effects of arginine and glucose on glucagon and insulin release from the perfused rat pancreas. J. Clin. Investig..

[37] Meijer A.J., Lamers W.H., Chamuleau R. (1990). Nitrogen metabolism and ornithine cycle function. Physiol. Rev..

[38] Suzuki T., Morita M., Kobayashi Y., Kamimura A. (2016). Oral l-citrulline supplementation enhances cycling time trial performance in healthy trained men: Double-blind randomized placebo-controlled 2-way crossover study. J. Int. Soc. Sports Nutr..

[39] Fernstrom J.D., Fernstrom M.H. (2006). Exercise, serum free tryptophan, and central fatigue. J. Nutr..

[40] Fernstrom J.D., Wurtman R.J. (1972). Brain serotonin content: Physiological regulation by plasma neutral amino acids. Science.

[41] Fernstrom J.D., Faller D.V. (1978). Neutral amino acids in the brain: Changes in response to food ingestion 1. J. Neurochem..

[42] Pardridge W.M. (1998). Blood-brain barrier carrier-mediated transport and brain metabolism of amino acids. Neurochem. Res..

[43] Sureda A., Córdova A., Ferrer M.D., Pérez G., Tur J.A., Pons A. (2010). L-citrulline-malate influence over branched chain amino acid utilization during exercise. Eur. J. Appl. Physiol..

